# Supplementary figures and images for: Prediction and analysis of novel key genes ITGAX, LAPTM5, SERPINE1 in clear cell renal cell carcinoma through bioinformatics analysis
Source: PeerJ. 2021 Apr 20;9:e11272. doi: 10.7717/peerj.11272 (PMC8063882; doi:10.7717/peerj.11272)

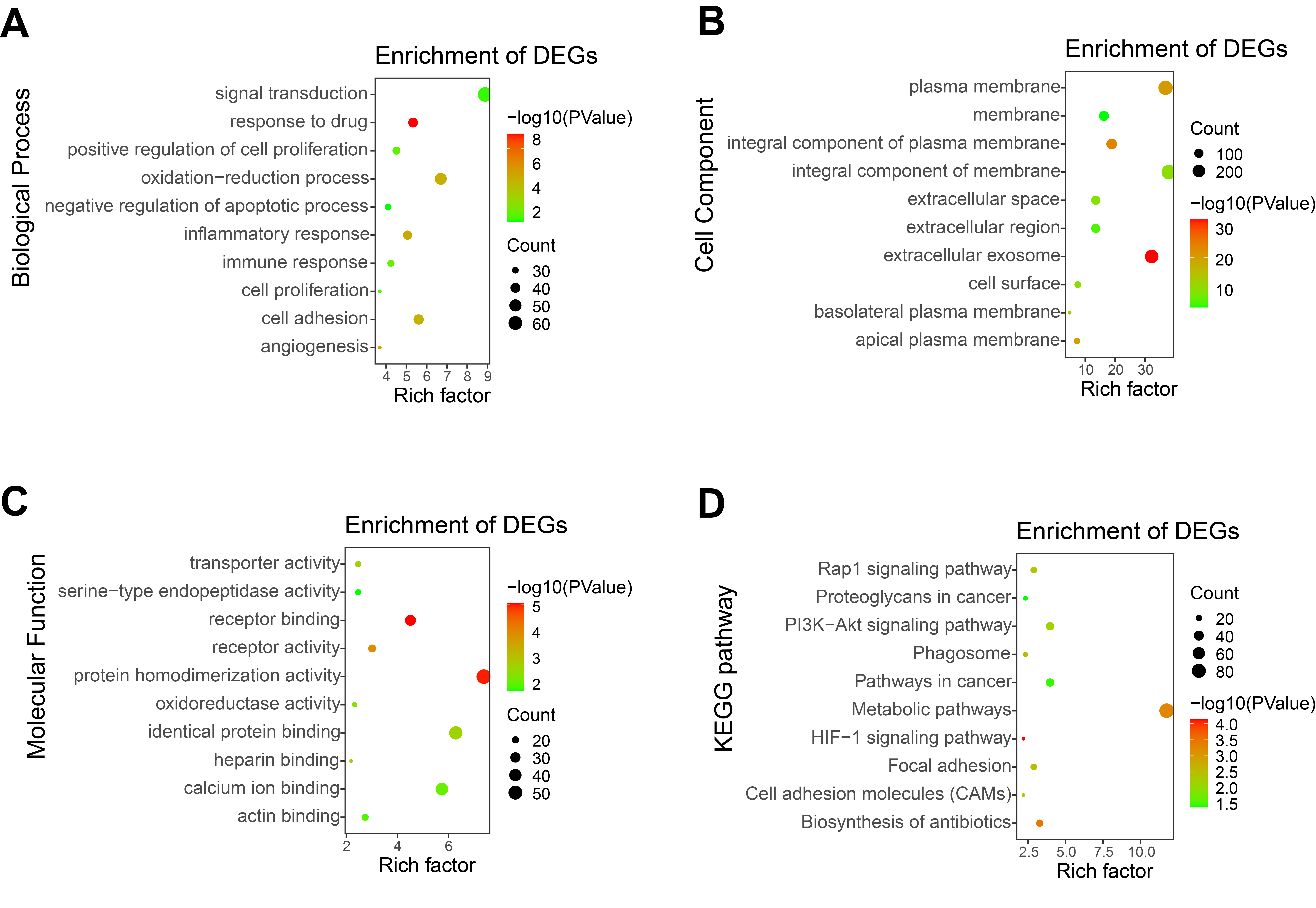

Supplement: Supplemental Information 5 — (A) Biological processes. (B)Cellular components. (C) Molecular functions (D) KEGG pathway. [file peerj-09-11272-s005.png]
